# Present Status and Future Growth of Advanced Maintenance Technology and Strategy in US Manufacturing

**Published:** 2016

**Authors:** Xiaoning Jin, Brian A. Weiss, David Siegel, Jay Lee

**Affiliations:** 1 Department of Mechanical and Industrial Engineering, Northeastern University, MA, 02115, USA; 2 National Institute of Standards and Technology, Gaithersburg, MD, 20899, USA; 3 Department of Mechanical & Materials Engineering, University of Cincinnati, USA

## Abstract

The goals of this paper are to 1) examine the current practices of diagnostics, prognostics, and maintenance employed by United States (U.S.) manufacturers to achieve productivity and quality targets and 2) to understand the present level of maintenance technologies and strategies that are being incorporated into these practices. A study is performed to contrast the impact of various industry-specific factors on the effectiveness and profitability of the implementation of prognostics and health management technologies, and maintenance strategies using both surveys and case studies on a sample of U.S. manufacturing firms ranging from small to mid-sized enterprises (SMEs) to large-sized manufacturing enterprises in various industries. The results obtained provide important insights on the different impacts of specific factors on the successful adoption of these technologies between SMEs and large manufacturing enterprises. The varying degrees of success with respect to current maintenance programs highlight the opportunity for larger manufacturers to improve maintenance practices and consider the use of advanced prognostics and health management (PHM) technology. This paper also provides the existing gaps, barriers, future trends, and roadmaps for manufacturing PHM technology and maintenance strategy.

## 1. Introduction

### 1.1. Overview

Reducing waste, improving equipment up-time, and optimizing product quality are three metrics important to manufacturing enterprises. Organizations have developed methods and metrics to measure their performance with respect to waste reduction, uptime, and quality to quantify their manufacturing performance. The most widely adopted metric by manufacturers is the Overall Equipment Effectiveness (OEE), which is used to evaluate the utilization rate or efficiency of factory equipment (Nakajima, 1988; Liker, 2014). Equipment and process health states are highly correlated to OEE, thus there is growing interest in developing intelligent maintenance systems to improve OEE, and predict and prevent unexpected equipment and process downtime.

The various maintenance strategies that manufacturers have deployed are in a constant state of evolution given the increasing complexity of manufacturing equipment and processes. Manufacturers use a combination of reactive maintenance (RM), preventive maintenance (PM), predictive maintenance (PdM), and proactive maintenance (PaM) to maintain their fleet of assets, in which the maintenance strategy for a given asset depends on the complexity of the machine and the impact an unexpected failure has on that machine. With improvements of Internet of things (IoT) augmented with computing power, sensors, network communication, and machine automation, real-time diagnostic and prognostic technologies become emerging research topics in various manufacturing sectors (Coble, et al., 2015; Samah et al., 2015; Lee et al., 2011). Despite the increased interest in prognostics and more advanced maintenance strategies, manufacturers lack a standard process and methodology for using prognostic and health management (PHM) technologies on the shop floor.

It is important to understand and define a common set of performance metrics for productivity, maintenance, and product quality that are being used by manufacturers to develop a methodology and standard for PHM technology for manufacturing. These metrics can quantitatively evaluate the effectiveness of diagnostic, prognostic, and intelligent maintenance activities when compared with other maintenance strategies. In addition, it is important to understand the best practices in industry for achieving their maintenance and productivity goals. Surveying various manufacturers can help determine these best practices as well as which strategies are less effective.

### 1.2. Research Objectives

The main objectives of this pilot study are the following:

- Identify the common metrics used by the manufacturing industry to assess their productivity, maintenance and reliability, and product quality.
- Investigate the best practices that manufacturers are using to improve their productivity, lower their maintenance costs, and improve their product quality.
- Assess the current states of the practice in the manufacturing sector with respect to diagnostic and prognostic activities, and review some past successes and failures.

Sections 3 and 4 present the outcomes of the aforementioned objectives in detail.

The information from the survey-based study will provide a strong foundation for developing a set of standards and a methodology for deploying intelligent maintenance systems technology across manufacturing applications. The results from this study could determine several important aspects, including 1) whether there is a statistical difference between the number of successful implementations of diagnostic activities for large manufacturers when compared with small to medium size enterprises (SME) manufacturers and 2) developing an understanding of the common challenges for manufacturers for implementing prognostic and diagnostic technology. The reporting of these key findings and statistical results would be imperative for understanding the current status and needs of the manufacturing industry. These results would be later used to develop appropriate standards for prognostic and diagnostic activities that address the identified needs in this survey.

### 1.3. State-of-the-Art Research on Maintenance Strategy and PHM

#### 1.3.1. Maintenance Strategy

Manufacturers employ a range of maintenance strategies to reduce waste, maximize equipment up-time, and optimize product quality. Maintenance strategies are also determined based upon available resources, including technology and personnel. Resource availability/limitations can ultimately be traced back to available finances. Manufacturers are seeking to optimize the amount of money they invest in their equipment, technology, and workforce to maximize their profit. Part of this optimization problem is to determine the most appropriate maintenance strategy for the many components, machines, work cells, and lines within the factory. Selecting the appropriate maintenance strategy(ies) is non-trivial where each strategy is unique with varying characteristics.

Table 1 presents the evolution and overview of maintenance strategy. Each maintenance strategy (or practice) has a variety of characteristics. They are described as:

- Maintenance Interval – The determination of when maintenance is conducted
- Object – The primary areas of focus of a particular maintenance strategy
- Planning & Scheduling – Strategy in which maintenance activities are planned and scheduled
- Human Factors (inspection & decision-making) – the overhead (i.e. cognitive and time demands) placed on operators, maintenance personnel, supervisors, etc. under the various maintenance strategies
- Cost Effectiveness – Projected/estimated cost of implementing the maintenance strategy
- Requirement for Technology Readiness – Necessity of advanced technology to enable a maintenance strategy

A brief discussion of each maintenance strategy (identified as the column headers of Table 1) is provided to highlight the advantages and disadvantages of each approach.

*Reactive maintenance* is a corrective action applied on observable failures. RM has a relatively low investment cost although cost increases typically arise from unscheduled equipment downtime and production losses. *Preventive maintenance* involves the repair, replacement, and/or maintenance of equipment at predetermined unit, cycle, or time interval to avoid unexpected failure during operation. The objective of any maintenance program is to minimize of the total cost of inspection, repair, and equipment downtime (measured in terms of lost production capacity or reduced product quality). A successful PM strategy that improves equipment availability has two drawbacks: 1) time-based or operation count-based PM programs lead to potentially over-maintained equipment, especially in instances when the PM interval is predetermined without considering various operational regime shifts; and 2) replacing the component before it severely degrades or fails does not allow for insightful information to be learned about the equipment’s lifecycle (Lee et al. 2013).

PM can become a major expense for many industrial companies. Therefore, more efficient maintenance approaches, such as predictive maintenance are being implemented. *PdM* is a right-on-time maintenance strategy. Predictive maintenance can be classified into reliability-centered maintenance (RCM) and condition-based maintenance (CBM). However, this maintenance strategy is more commonly implemented as CBM in most production systems where certain performance indices are periodically (Barbera et al. 1996; Chen et al. 2002) or continuously monitored (Marseguerra et al. 2002). CBM is a technique or a process for monitoring the operating characteristics of processes and machines (or components). Changes and trends in the monitored characteristics can be used to predict the need for maintenance before serious deterioration or breakdown occurs. Thus, CBM attempts to avoid unnecessary maintenance tasks by taking maintenance actions only when there is evidence of abnormal behavior in a process or machine. By reducing the number of unnecessary scheduled preventive maintenance operations, a properly established and effectively implemented CBM program can significantly reduce maintenance costs (Jardine et al. 2006, Mann et al. 1995). For example, based on a high-level analysis of the automotive industry, Barajas et al. (2008) stated that the best return on investment is achieved through predictive maintenance as opposed to reactive or preventive maintenance.

*Proactive maintenance* focuses on understanding the failure modes, detecting precursors to failure, tracking degradation mechanisms, and predicting the remaining useful life of components, systems, and processes. Proactive maintenance commissions corrective actions aimed at the sources of failure. It is designed to extend the life of mechanical machinery as opposed to 1) making repairs when often nothing is broken, 2) accommodating failure as routine and normal, and 3) preempting crisis failure maintenance.

The decisions to implement an appropriate maintenance program must be based on the probability and magnitude of the failure along with the associated costs and consequences. Designing an effective and efficient maintenance strategy requires engineering efforts that optimize the relationship between equipment ownership and operating profits by balancing the cost of maintenance with the cost of equipment degradation and failures, and resultant production losses. PdM and PaM usually require an initial higher maintenance investment due to higher requirement for technology readiness, but can substantially save unnecessary failures, extend the life of equipment more so than simple RM and PM, and further minimize bad part/product generation.

#### 1.3.2. Manufacturing PHM

*Prognostics and Health Management* refers to a set of technologies that link studies of failure mechanisms to system lifecycle management. Specifically, PHM includes health monitoring, diagnostics, prognostics, and maintenance techniques. PHM can be used to determine the root causes of failures, predict degradation trends, and support decisions for optimal maintenance schedules to eliminate the sources of failure before problems occur. With an effective use of PHM technologies, maintenance can be planned more proactively and thus reduce unplanned downtime, unnecessary maintenance activities, and labor cost.

Manufacturing PHM research can be divided into machine-level and system-level studies. Much of the machine-level research has focused on machine tools, and this includes prior work on machine tool spindles (Cao et al., 2012; Vogl et al., 2015-1), cutting tool wear or breakage (Aliustaoglu et al. 2009; Amer et al. 2007, Malekian et al. 2009), and machine tool feed-axis systems (Liao et at. 2012; Sztendel et al. 2012; Vogl et al., 2015-2; Zhou et al. 2011). For machine tool PHM applications, the algorithms used by researchers include both data-driven and first-principal methods. For data-driven methods, a common approach includes the use of a classification method after features were extracted from the various signals; support vector machines, self-organizing maps, and variations on neural networks were some of the classification methods used in these machine tool case studies, respectively (Malekian et al. 2009; Liao et at. 2012; Demetgul, 2013). First principles methods include the work by Cao et al. (2012), in which a physical model of the spindle was used to determine the optimal sensor location for monitoring the health state of the spindle.

Industrial robot health monitoring is another popular machine-level monitoring application for PHM manufacturing research studies. First principle approaches that model the kinematics and dynamics of a particular type of robot were considered by Brambilla et al. (2008) and Liu et al. (2005), in which a residual-based diagnostic can be made by comparing the actual and predicted sensor responses. A data-driven approach that compares the robot joint angle speed and joint angle torque from a baseline condition using principal component monitoring statistics was conducted by Sjöstrand et al. (2010). This work used a variety of signal measurements for robot health monitoring, such as axis speed, axis torque, motor temperature, gearbox temperature, and calculated quantities such as cycle time and energy consumption.

The topic of validation is a challenge for many PHM studies including manufacturing applications. A common approach in the literature is to use machine (Aliustaoglu, et al., 2009; Amer et al., 2007; Malekian et al., 2009, Sztendel et al., 2012) or subsystem or component testbeds for generating data sets to help validate PHM methods and algorithms (Vogl, et al. 2015). Testbeds provide a controlled environment that allows one to introduce various failure modes at a controlled severity level. Data from the factory floor is less frequently used to develop and validate PHM health models in the literature. This approach is reasonable given that there are many uncontrolled factors in the factory environment that would make the validation aspect more difficult to accomplish. The National Institute of Standards and Technology (NIST) is actively developing numerous testbeds, including platforms at the component, work cell, and system levels, to support verification and validation of PHM methods and techniques (Helu & Hedberg, 2015; Weiss et al., 2015).

Although the majority of the work is conducted in controlled settings, some prior research work used factory data for developing their health models. The work of Skritich (2012) used various statistical anomaly detection methods for monitoring several machine tools used in a production setting. In this study, an axis pulley failure occurred on one of the machine tools; this problem could have been detected several days earlier using the proposed monitoring approach.

For system-level PHM manufacturing applications, the work conducted by Muthiah et al. (2008) introduced a new metric called the *overall throughput effectiveness*, which can provide a way to benchmark the factories current performance with respect to a baseline value. In addition to providing a way to monitor factory performance, this proposed metric could also be used for detecting factory bottlenecks, which are important in diagnosing the root-cause in a drop in factory performance. For conventional metrics, such as OEE, its success depends on the ease of collecting the data and enabling operators and plant personnel to visualize the information. Given the overwhelming majority of the prior work on machine-level PHM applications in comparison to system-level PHM applications, there appears to be a research gap on system-level PHM research methods and techniques that should be addressed. In addition, even the PHM machine-level methods would be aided by common data sets for improving and validating their methods and algorithms; generating PHM manufacturing benchmarking data sets should be considered as a future direction for manufacturing PHM research.

## 2. Methodology

### 2.1. Survey Questionnaire Development

Research personnel from the University of Cincinnati (UC) and the University of Michigan (UM) – Ann Arbor performed pilot surveys and case studies in 2015. The data were solicited via emailed questionnaires followed by phone interviews and onsite visits of 23 selected U.S. companies. The survey questionnaire was formulated to cover a broad range of manufacturing industry sectors. The questions were based on different perspectives of maintenance practices and contained six categories of questions: (1) manufacturing system performance measurement, (2) diagnostics and prognostics technology, (3) maintenance strategy and effectiveness, (4) key factors that affect maintenance performance, (5) future trends for PHM technology for smart manufacturing from an industrial perspective, and (6) challenges and future plan for intelligent maintenance technology.

Sample data were solicited by the UC/UM team via questionnaires, phone interviews, and on-site facility visits with a variety of manufacturing enterprises ranging in size. The focus of the survey and interviews was on manufacturing managers, maintenance managers and engineers, and other senior professionals within the production and maintenance function. A total of fifteen (15) manufacturers ranging in type and size and eight (8) technology/consulting companies provided responses to the questions through surveys and interviews. Table 2 summarizes the profile of the respondents. The manufacturing enterprises provided the most direct responses to the questions based on their own maintenance strategy, operations and practices in PHM development and implementation, while the technology/consulting companies provided more comprehensive information such as common PHM solutions to various types of industrial sectors. The enterprises represent various sectors within manufacturing, including: automotive, aerospace, transportation, machinery and equipment, consumer products, and electronics.

Respondent feedback was based upon individual’s own daily observations and estimations. Although the use of objective measures would have been more desirable, it has been difficult to acquire exact data for a variety of reasons such as limited data collection capability, confidentiality, and accounting conventions.

By analyzing the responses from the survey and summarizing the present status of manufacturing enterprises in diagnostics/prognostics technology and maintenance strategy, this paper undertakes exploratory work in this area to address numerous questions including:

- What are the commonly used maintenance objectives and performance metrics for manufacturing enterprises?
- Which factors influence the selection of maintenance strategy and its effectiveness?
- Are the manufacturers willing to improve their maintenance technologies and strategies? Which factors are the barriers for manufacturing enterprises to improve their maintenance strategies?

### 2.2. Open-Ended Discussions

Personnel from NIST conducted case studies complementary to the efforts of the UC/UM team. NIST personnel organized their case studies to better understand existing manufacturing processes and operations including the investigation of high-value challenges, fault/failure modes, and bottlenecks for processes and equipment. Case study engagement began with a brief phone conversation so that the study’s goals could be presented, and any potential concerns shared. NIST personnel then conducted a site visit of the participants’ facility that featured discussions and a tour. The conversations evolved according to the participants’ preferences. Given UC/UM’s focus on large manufacturers, NIST focused the case studies on SME manufacturers, technology providers/integrators, and consulting enterprises. Space limitations restricted discussion of all of NIST case studies in this paper; NIST personnel spoke to representatives from ten different SME organizations. Three case studies conducted by NIST are presented in this paper: two represent manufacturers and one represents a technology provider. All case study participation was voluntary.

## 3. Case Studies and Insights

### 3.1. Large-Sized Manufacturing Enterprises

#### Case Study 1

During the observational studies and site visits to various manufacturers, one particular transportation manufacturer’s initiatives on predictive and proactive maintenance technologies are worth highlighting. At the time of the survey, this rolling stock transportation manufacturer was in their first year of implementing new technology at their remanufacturing facility, in which they have approximately 30 different machines, including machine tools and washer equipment. It should be noted that the factory could be considered a pilot factory for the company considering PHM technology.

As data is one of the critical bottlenecks for developing more predictive maintenance practices, this manufacturer has put many significant efforts in the past year to develop a data collection infrastructure and sensor strategy for various machines. In particular, their washer equipment has received added instrumentation and sensing, including flow rate, temperature, pressure, and conductivity measurements. This organization is also in the process of instrumenting the machine tools; a deliberate pace is being set to ensure the appropriate strategy is focused upon that will align with a proper cost-to-benefit ratio for the machine tools. Less critical equipment in the plant will not obtain the same level of sensing and monitoring but would be upgraded to pull out information about the operational status of the machine. This operational status information would help facilitate OEE calculations and provide a more tractable way of comparing and trending the current plants performance.

Although this is a rather significant investment in resources for monitoring their manufacturing equipment, the organization has already obtained some early success from monitoring one of the critical washer machines in their remanufacturing facility. For this particular machine, they have had three different alarming events, in which they had an early indication of an impending failure that was correctly detected and corrected before any costly downtime or failure occurred. Although the current detection method was a combination of statistical process control and visual inspection of the sensor signals, the goal is to automate this analysis in the future. The organization’s plan is to leverage their company’s existing portfolio of data analytics and anomaly detection algorithms to automate this process as they collect more data and have a better baseline fingerprint for each machine.

#### Case Study 2

A case study visit with an aerospace manufacturer provided another insightful perspective on some of the barriers for achieving success with implementing this PHM technology. This manufacturing facility performs assembly of aviation systems, and consists of machine tool and quality inspection equipment. In one of their past monitoring examples, the organization developed an early warning (anomaly detection) system that had a detection accuracy with a low false alarm rate. From a technology perspective, this early warning system was a success; however, there were additional challenges that resulted in this monitoring system not providing the value that was expected. In particular, the operators ignored the early warning system even though the early warning system was accurate in providing a correct detection. It is hard to conclude why the operators ignored this warning system, yet it shows that there are significant work cultural barriers for implementing PHM technology within a manufacturing facility.

This same aerospace manufacturing facility also had a manufacturing PHM case study with machine tools that highlighted some additional challenges for achieving successful implementation of this technology. During a pilot research study, they developed a machine tool health monitoring system, in which various controller parameters were collected and monitored during a routine machine motion profile/test that was conducted once per shift or day. Various analytics and multivariate statistical tools were then applied to the collected data to generate a health metric. In turn, this metric was used to estimate the health condition of the various machine tool subsystems and components over time.

During the pilot study, the developed analytics were evaluated against historical data and the results showed that this approach could provide an early detection of a failure with one of the machine tool axes. After the pilot study, the solution was deployed and used to monitor a set of machine tools over a one-year time-period. However, during this time-period, no failures occurred. Considering that the routine test took time away from production and that no failures occurred since the solution was implemented, they eliminated the routine test and monitoring solution. However, after this monitoring was discontinued, a similar axes failure occurred on one of the machine tools and they are now considering renewing this monitoring solution.

This chain of events highlights the challenge that many PHM solutions could face, in that they will have to overcome short-term performance and evaluation metrics. For many manufacturing equipment, it might be difficult to justify PHM solutions in the short-term given that many components degrade over time and failures would likely not occur in the short-term.

#### Case Study 3

A large consumer products manufacturer has been actively developing and adopting its own PHM and maintenance optimization methodologies. The manufacturer’s methodology goes beyond the traditional fail-and-fix maintenance mode, and uses to high-end engineering with predictive capabilities and an uptime vs. downtime focus. The main challenges for the manufacturer to implement new PHM technologies and preventive maintenance strategies are mainly attributable to the unique characteristics of the high-throughput, high-speed production systems. Yet fail-and-fix is a costly option. Stoppages due to machine breakdown could significantly reduce the OEE, and cause hundreds of defects during the restart/transient period. Hence, the goal of the manufacturer is to improve the OEE ratios to be at or exceed 95 %, and reduce the downtime-induced defect.

This manufacturer has realized that, although the adoption of lean and sigma programs creates greater awareness that optimized production pays big dividends in output and quality, the production speed and efficiency are not sufficient enough to compete globally. The manufacturer envisions that a set of intelligent maintenance tools and PHM technologies need be adopted within their manufacturing facilities in the near future. These technologies include smart sensors found on automated controls, remote monitoring systems, and software applications that can detect and diagnose problems – even remotely – or generate repair orders directly to maintenance management system. The manufacturing system would also need other specialized machines to monitor key operational indicators such as abnormal hot spots, leaks, and vibration problems.

The challenge of investing in resources for new sensing, diagnostic, and analysis technologies has more to do with motivation rather than money. The first step for the company to move from a fail-and-fix mode to a preventive and/or predictive mode is to create a culture of change that moves from passive to proactive maintenance. The next step is to update operators and maintenance technicians with the expertise to run the latest computerized monitoring tools and devices. To address these challenges, additional incentives for interdepartmental collaboration and research and development (R&D) development will be needed.

### 3.2. Small to Mid-Sized Manufacturing Enterprises

Three SME case studies are presented in this section. Perspectives from two manufacturers and one technology integrator are specifically highlighted.

#### Case Study 4

The first SME case study participant is a relatively small original equipment manufacturer (OEM) who is responsible for manufacturing a component of varying sizes for their parent company. This OEM produces these components using a combination of sheet-metal forming, machining, and welding processes. These components are predominantly used in the shipping, chemical, pharmaceutical, and food industries. The company currently employs a little less than 100 people where approximately 75 % work on the shop floor. This organization delivered nearly 20,000 parts in 2015 where it typically takes two to four weeks to fabricate a single part. At any given moment, the shop has between 400 to 600 orders as work in progress (WIP). The company’s current enterprise resource planning software is SAP (Karnouskos et al. 2010; Giriraj and Muthu, 2010) whereas their previous solution was custom-made to handle day-to-day operations. The transition was expensive for the company and challenging – there was little experience with SAP and it did not integrate well with the existing systems.

For this organization, it is critical they measure and track conformance to original cost estimate, expected time of delivery, and part specification. Their maintenance strategy is driven by the high operation and tooling costs, and challenges faced when ordering spare parts (i.e., they are not readily available). Preventative maintenance is the dominant strategy, yet reactive maintenance still occurs if/when machines unexpectedly fail. Most of these failures are typically tied to bearing issues. To minimize this downtime, the company has invested in a spare parts inventory, which is kept in an adjacent warehouse. The company is very interested in transitioning to a predictive maintenance strategy. With the high demand of their machines, the hope is that a predictive maintenance strategy will enhance the ability to plan necessary maintenance around critical machining operations with both minimal downtime and minimal financial impact.

To achieve a predictive maintenance strategy, the company is taking the very critical step of transitioning from manual (their current modus operandi) to paperless data collection. Data collection is challenged, and the transition will be challenged, by the large WIP. To support this transition, the company is exploring networking equipment and systems, cloud-based services for data storage, and job tracking systems to determine status and improve scheduling. Significant concerns exist in making this transition including expected difficulties with integrating solutions across heterogeneous systems, lack of sufficient data to support analysis and decision-making (i.e., there is not paperless baseline data with which to draw upon), and the disruptions to daily operations that can occur with such a transition.

#### Case Study 5

The second SME case study participant can be described as a larger-scale contract shop that specializes in large work volumes. This shop is predominantly focused on computer numerical control (CNC) machining. The produced parts and components support the chemical processing, energy, mining, machine tool, aerospace, paper, plastic, and steel industries. Two of this SME’s key metrics are basic utilization (in-cycle versus not-in-cycle), and start-time versus in-cycle. The company has thousands of part numbers (in total) where approximately 600 are currently under contract. Low volumes are typically requested when a specific part is produced. Standardization of software packages, machine tools, and controllers is sought as challenges when working with heterogeneous systems and interfaces.

Given the company’s high burn rate^1^ for equipment, successful maintenance and scheduling strategies are required to minimize nonproductive times. Job estimates (including cost and resource allocations) are based upon tribal knowledge, yet there has been a historic trend of underestimating when comparisons are made to actuals, which has a negative impact on burn rate. Another job scheduling issue is that jobs are not planned beyond 2+ weeks of operation. A significant challenge to enhancing their maintenance strategy is the lack of staff that are open to and support modernization and emerging technology. The company’s current maintenance strategy is largely reactive where prior efforts to introduce preventative maintenance were met with heavy resistance from the staff due to culture. Another attempt is being made to implement preventative maintenance.

Presently, unexpected breakdowns occur every few days which usually take, on average, over a day to resolve. Unexpected issues have been more prevalent in the summer when temperatures are above normal during operations. Given these factors, the organization is also interested in condition-based maintenance where active health monitoring can better inform personnel of when and where maintenance should occur. As such, support is increasing for real-time supervisory monitoring and control of shop-floor operations along with dynamic scheduling. In addition to the cultural challenges of advancing their maintenance strategy, the company also faces a lack of sufficient data to support equipment health analysis. Low volume part runs have made it difficult to learn much from current operations so data collection efforts have mostly targeted work centers that maintain relatively consistent operations. Similar to the Case Study 4, this organization is also challenged by the heterogonous systems mix given their lack of common interfaces and licensing issues. Another challenge is with respect to communications given specific cyber security requirements. These requirements are usually imposed internally to maintain the integrity of the data and the overall manufacturing operations.

### 3.3. Technology Providers/Consulting Enterprises

#### Case Study 6

One case study participant is a SME technology integrator; they are contracted by manufacturing customers to provide various solutions to enable specific manufacturing capabilities, typically in the form of developing and deploying new work cells. These work cells often blend industrial arm robotics, programmable logic controllers, networking equipment, and other automation technologies to complete a specific process. This integrator is not tied to any specific industry, and has provided work cell solutions to a range of organizations including those in power tools, medical devices, and food/beverage packaging.

In creating work cell solutions, the integrator noted it is very rare for their customers to ask for specific diagnostic and prognostic techniques; rather, they have specific productivity, performance, and quality requirements that the integrator must target. Any maintenance information that is provided to the customer comes from the manufacturers of the individual pieces of equipment (e.g., embedded sensors, robotics, and safety systems) that the integrator builds in to create the work cell. Two example work cells that the integrator recently developed incorporate multiple industrial robotic arms, and feature a majority of the work cell secured behind a fence. Minimal diagnostic and prognostic capabilities are integrated into these work cells because these were not requested by the customers. It is typical for the customer to not specify any diagnostic or prognostic capabilities because they are either unaware of such capabilities, or they recognize that this will increase the cost of the solution. However, the customer requirements typically dictate that the integrator is responsible for designing and creating the human-machine interfaces. These interfaces provide the operators with the necessary controls and information to operate the machines effectively and safely. Since the integrator’s solutions are motivated by the customer’s requirements, there is little motivation for the integrator to add/enhance their PHM capabilities in their engineering solutions if the customer is not requesting it.

## 4. Results, Analysis, and Discussion

### 4.1. Preliminary Observations

Based on the survey and case studies, we summarize the maintenance objectives across the various manufacturing enterprises and technology/consulting companies and classify the commonly used maintenance performance measures into six categories:

1. Equipment performance (e.g., availability, reliability, mean time to failure),
2. Product quality performance (e.g., defect rate, yield),
3. Maintenance productivity performance (e.g., manpower utilization, efficiency),
4. Maintenance cost (e.g., maintenance labor and material cost),
5. Safety and environment (e.g., safety, health and environment incidents), and
6. Production/process performance (e.g., work-in-process, cycle time).

#### 4.1.1. Survey Results and Analysis

The key findings according to the respondent’s selection of important objectives are the following: (1) safety (92 %) and (2) availability and reliability (77 %) are the top two highly rated maintenance objectives. Productivity and quality are equally important (69 %) to the manufacturers because they directly affect the cost-effectiveness of their production systems. Typically, manufacturing organizations use performance metrics to measure system-level performance such as productivity, maintenance, product quality, or a combination of these metrics. The majority of the manufacturer surveys draw both statistically significant conclusions on individual metrics (e.g., throughput, defective parts per hour, and maintenance-related metrics) and a combination of these metrics. This is well aligned with the trend of companies adopting OEE metrics to measure for factory performance monitoring and evaluation (Jonsson and Lesshammar, 1999; Liker, 2014).

**Figure 1:**
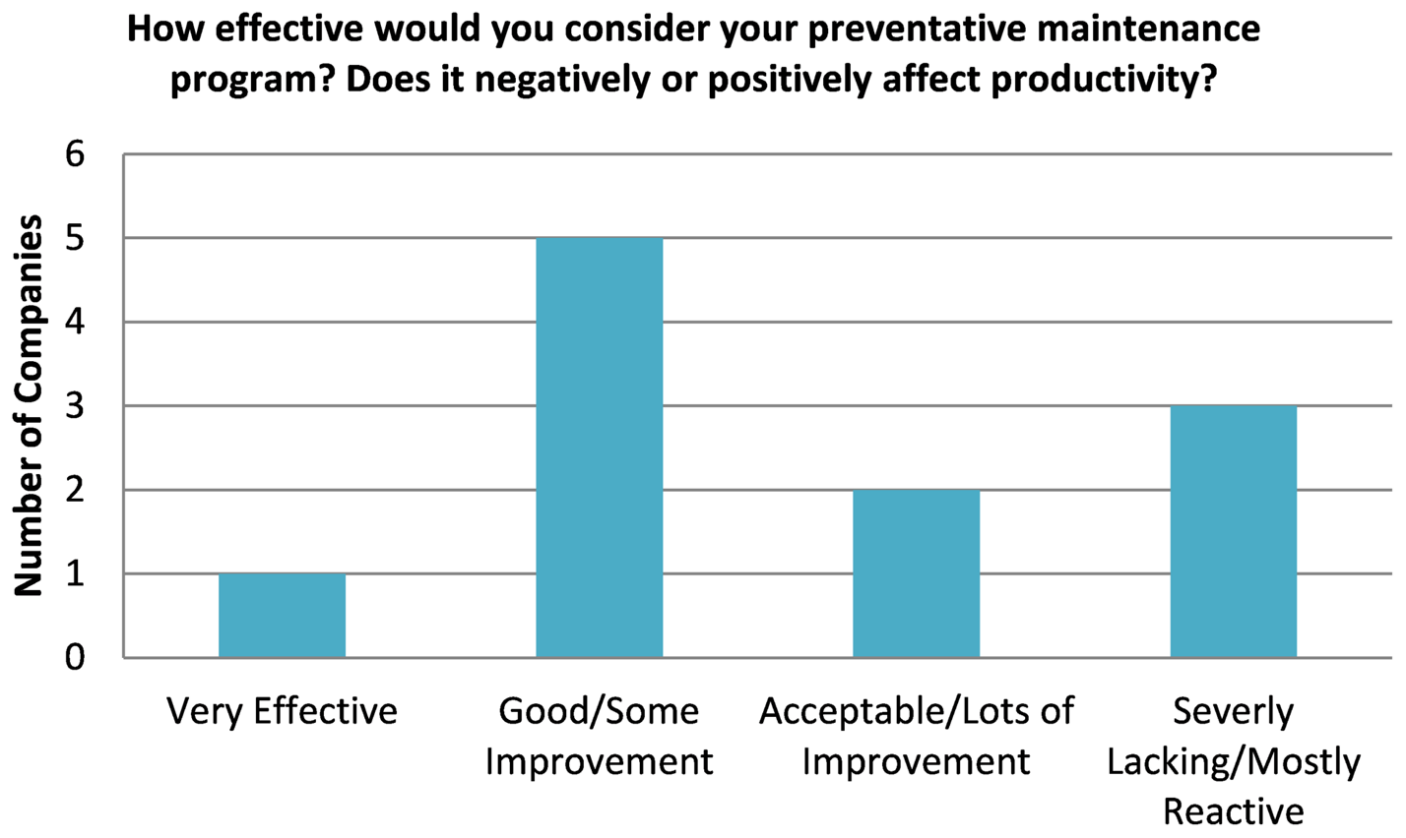
Manufacturers – Preventative Maintenance Effectiveness Survey Response

For maintenance strategy, it was important to have a grasp on the manufacturers’ current maintenance practices and whether these practices are effective, or if they have room for improvement. This provides some measure of where manufacturing organizations are in terms of maintenance strategy and this could influence their future adoption of more advanced maintenance technologies. For preventative maintenance effectiveness, the response from the manufacturers is provided in Figure 1. The noted level of effectiveness was diverse among the surveyed manufacturers. The general observations from the surveyed companies were that some of the larger manufacturing facilities had a more effective preventative maintenance program. However, this would also depend on the diversity of assets that they had in their facilities along with the age of the factory and equipment. For some of the smaller manufacturing facilities visited during this study, reactive maintenance (instead of preventative maintenance) was noted as their current strategy.

Some important insights were also gained on whether condition-based maintenance (CBM) strategies for certain types of machines or processes had been considered by manufacturing organizations surveyed in this study. A vast majority of organizations (72.7% of the survey manufacturers) are considering condition-based maintenance (CBM) approaches. We performed a Chi-square statistical hypothesis test in the following general form of equations (1) and (2) to provide statistical evidence that manufacturing organization are starting to consider and move towards CBM strategies. Chi-square test is used to investigate the “goodness-of-fit” between the observed and expected. The test statistic χ^2^ is defined as equation (1) with degree of freedom (*df*) of *n*-1, where *n* is the number of observations. The test-statistic and p-value with a significance level of α = 0.05 indicate that there is evidence that the responses are not random and the null hypothesis is considered as true.

(1) $$\chi^{2}=\sum_{i=1}^{n}\left(\frac{{\left({\mathrm{Observed}}_{i}-{\mathrm{Expected}}_{i}\right)}^{2}}{{\mathrm{Expected}}_{i}}\right)$$

(2) df=n-1

We further investigated whether the manufacturers who have started CBM had past and/or active projects in manufacturing diagnostics and prognostics. A Chi-square test is a statistical test procedure for categorical variables consists of comparing the expected bin frequencies to the observed bin frequencies. Based on the hypothesis that the responses are random, one would assume an expected frequency count that was even for each bin group. Test results reveal that manufacturing organizations are starting to move towards CBM strategies although the sample size is relatively small. More test details can be found in a prior publication (Jin et al. 2016).

**Figure 2:**
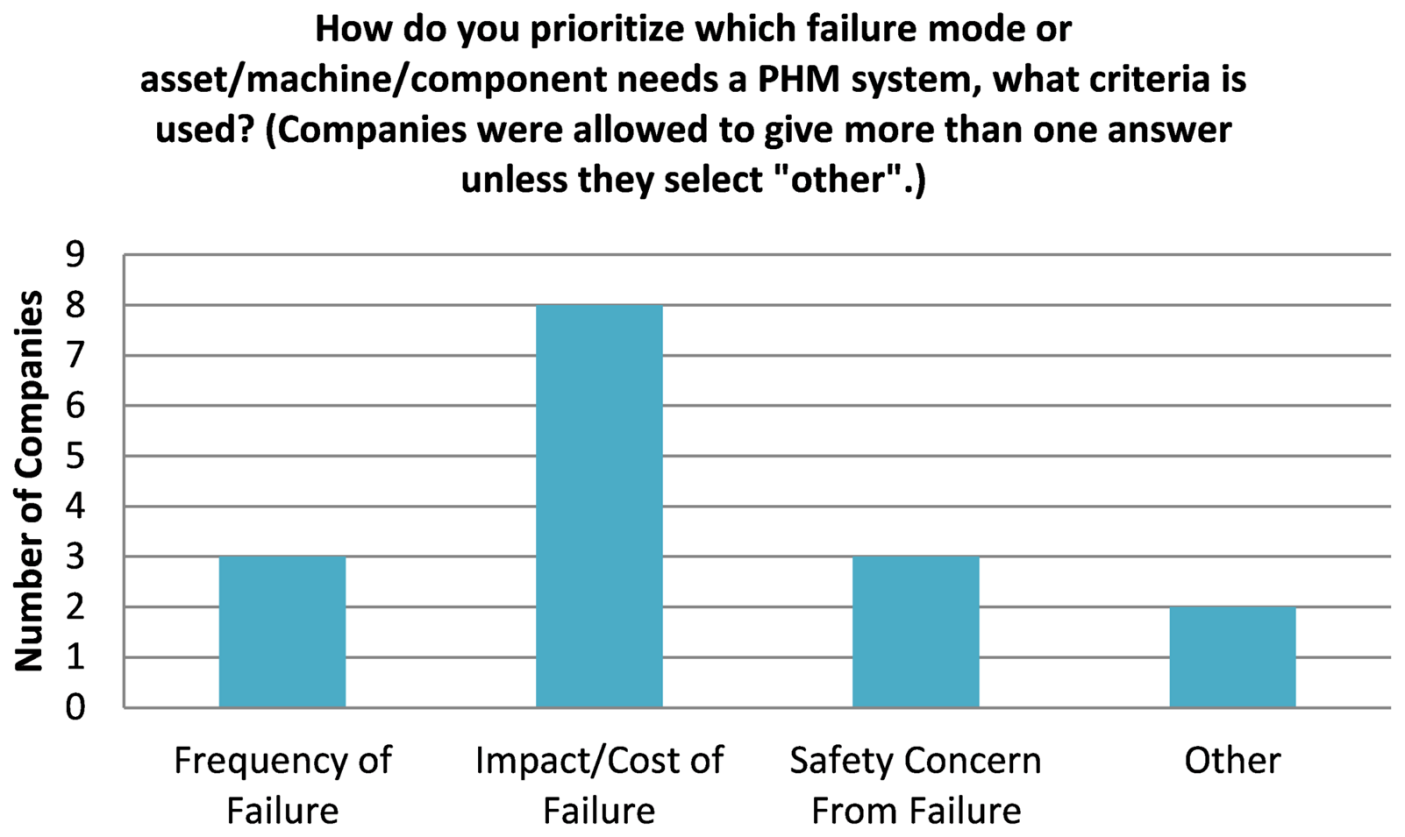
Technology Providers – Failure Mode/Criticality Analysis Survey Response

One of the interesting questions that was asked to both the surveyed technology providers and manufacturers is how does one determine which machine(s) or process(es) has the greatest need for a prognostics and health management system. Determining which machine is important and which failure mode to target can help determine the value and return on investment that a PHM system provides to the manufacturing organization. The response from the surveyed technology providers is highlighted here, since a technology provider might have a more diverse perspective if one considers that they work with multiple manufacturing organizations. The responses from the technology providers in Figure 2 indicate that the majority of them consider the impact/cost of failure as a key criterion for ranking machines and failure modes. The frequency of failure was also mentioned, although some mentioned that the frequency of failure might be misleading if the cost of the failure or downtime is low for that failure mode.

A Chi-square hypothesis was also performed for this response and the results in Table 3 indicate that there was not significant evidence to reject the null hypothesis that the responses were random. Perhaps there is not an overwhelming consensus on which criterion for failure mode ranking and criticality should be used.

**Figure 3:**
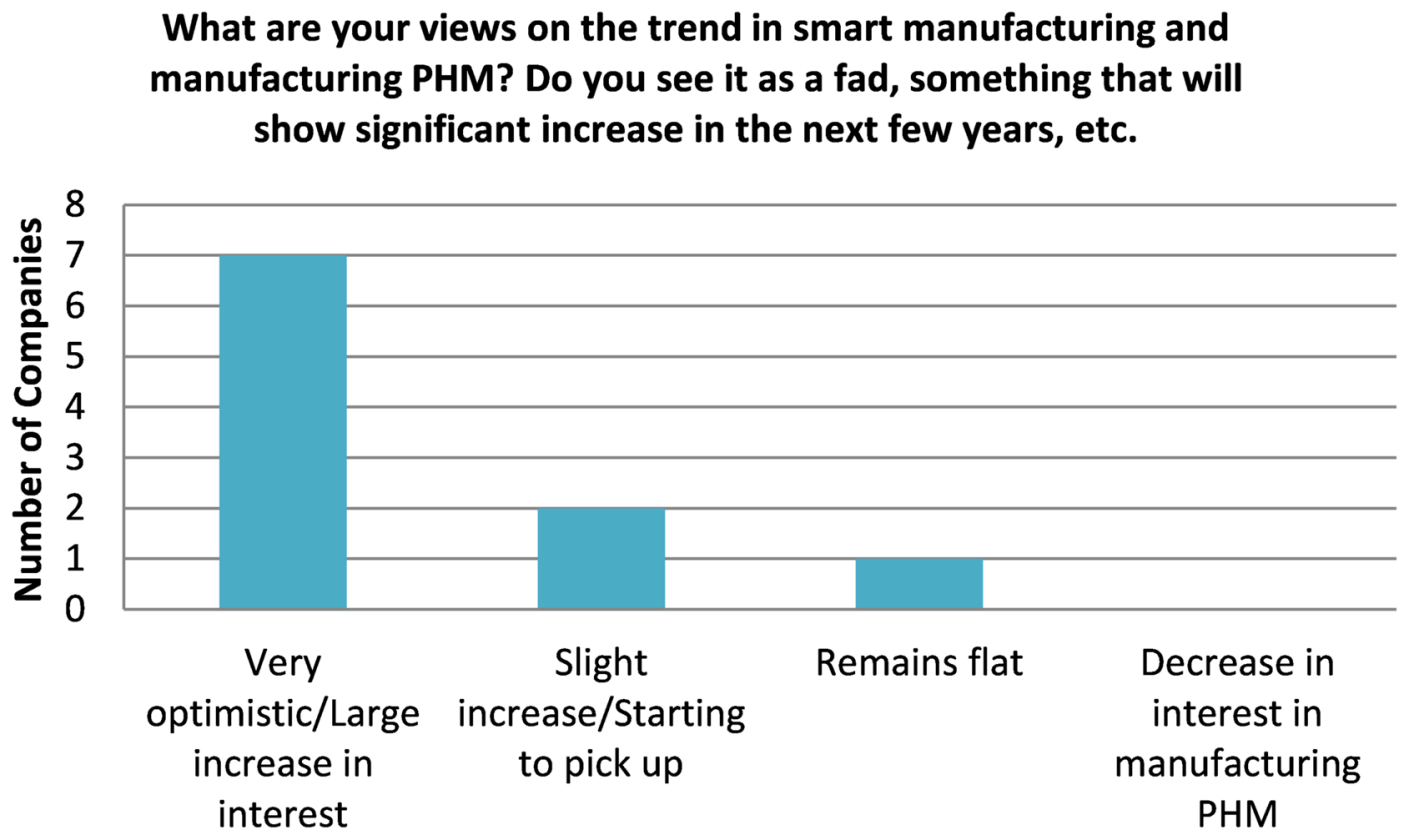
Manufacturing PHM Trend Optimism - Survey Response

One of the other important elements to gain some observations during this study was the perspective of manufacturers and technology providers on the future outlook for manufacturing PHM. With respect to the technology providers, the majority had a very optimistic view on manufacturing PHM (Figure 3). A few thought manufacturing PHM would have a slight increase or remain flat, while the vast majority felt that it would have a large increase in the next few years. The Chi-square test results in Table 4 also highlight that there was sufficient evidence to reject the null hypothesis and the responses appear to favor the optimistic viewpoint. One interesting comment was that there was past precedence within manufacturing to adopt trends from some leading manufacturing organizations, such as lean manufacturing. The rationale was that a similar trend would occur for manufacturing PHM, once a few leading manufacturing organizations had successful demonstrations of PHM systems and could highlight the value and cost savings.

**Figure 4:**
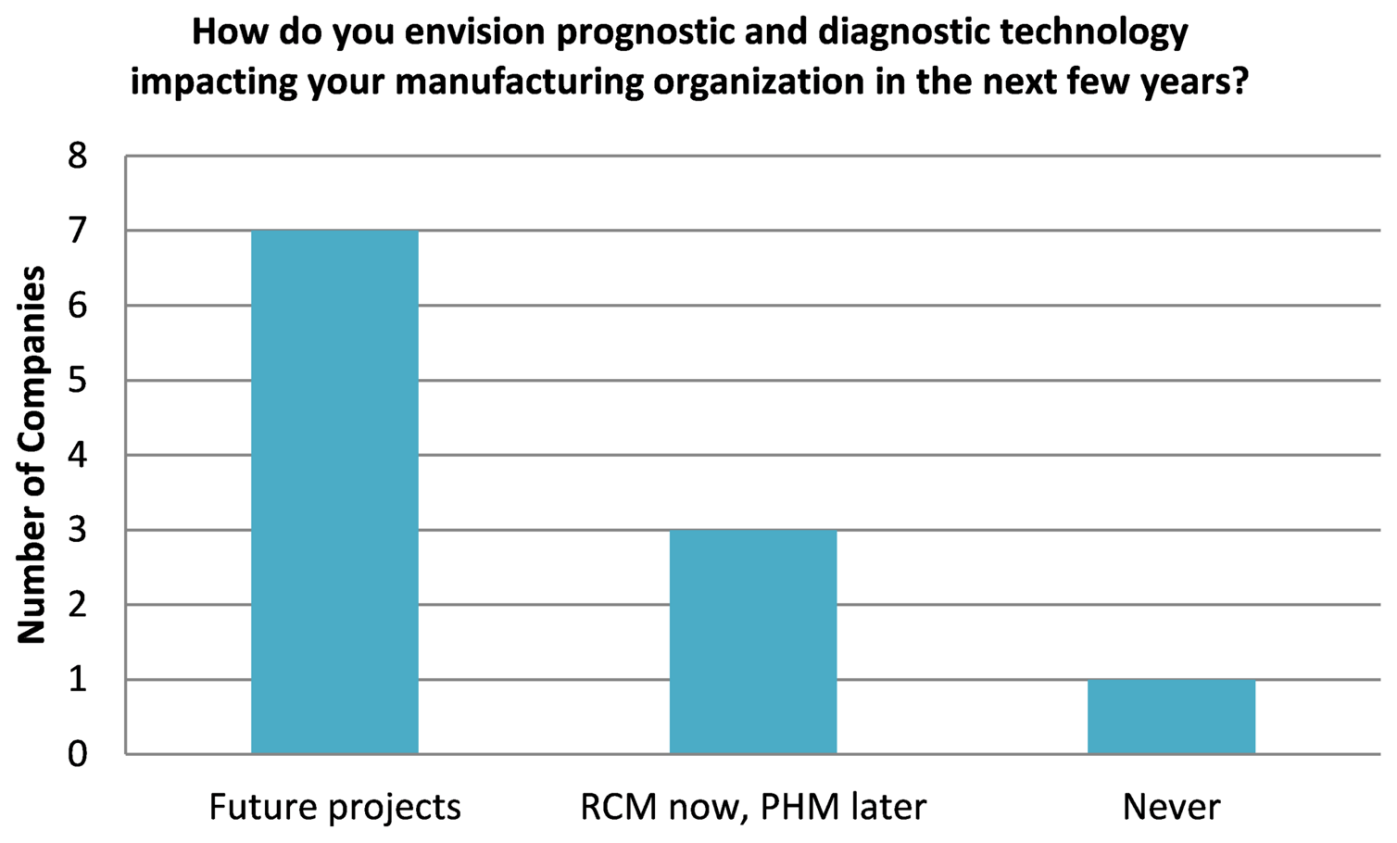
Manufacturers -Prognostic/Diagnostic Future Outlook - Survey Response

Although the technology providers had a very optimistic viewpoint on the future trend for manufacturing PHM, it was also important to see if the same sentiment was obtained from the manufacturers. The manufacturers would be the ones that would ultimately deploy and use this technology on their manufacturing floor and their level of optimism for manufacturing PHM might be a better gauge for the future trend and outlook in this technology area. The responses in Figure 4 indicate that many manufacturers have planned future diagnostic and prognostic projects, while only a few are just focused on RCM with no future PHM projects on the horizon. With the vast majority of manufacturers having future projects planned in this area, the manufacturers also appear to be optimistic about manufacturing PHM.

#### 4.1.2. Case Study Findings of SMEs and Technology Providers

NIST’s discussions with SMEs and technology providers were very insightful. From the SME perspective, this category of manufacturers is typically limited in their equipment and computing resources investment unless they can clearly justify the cost(s) and reasonably estimate the pay-back period of such investments. In most instances, this holds true for investing in PHM technology. These expenditures may be risky to a SME’s survival. If the investment yielded or exceeded the expected returns, then the company increases its overall health and profitability where further growth can be achieved. On the other hand, if the expected financial returns are not met, a SME may be faced with tough decisions in terms of cutting its workforce or even closing its doors.

Technology providers are in a different position with respect to investing in PHM technologies. Their technology development and implementation is motivated by their customer’s requirements. In this case, the manufacturer is the customer where a significant percentage of the technology providers’ customer base is from the SME community. Technology providers will add technology/feature enhancements to their solutions if it is a financially sound decision (i.e., if the customer is willing to pay for it, then the technology provider will do it). Unless technology providers are aware of emerging and advanced PHM technologies, and can highlight their value to the manufacturing customers or manufacturers have a direct appreciation of PHM, the PHM technology advancement within the SME community will be very limited.

Another insight that was discovered is that culture has a tremendous impact on how the overall organization perceives advanced and emerging technologies that are intended to augment an operator’s knowledge and capability at the shop floor level. PHM is one such technology where some SME operators viewed it with distrust or skepticism. In this case, the operators viewed the technology as supplanting some of their responsibilities (e.g., “the technology is doing something that I am equipped to do”) or the technology was not trusted to provide accurate information (e.g., false alarms). Several SMEs highlighted how culture played a significant role in how easily PHM was embraced or they noted how a resistant culture forced upper management to revise their PHM deployment strategy.

A strong commonality among the SMEs that were surveyed was that they are all subjected to reactive maintenance (i.e., as much as they tried to prevent failures, they still occurred). However, all took steps to balance this out with limited preventive maintenance strategies. Very few SMEs surveyed employed predictive maintenance approaches. Any predictive maintenance that was performed is very limited in scope for a SME. None of the SMEs that participated presented end-to-end predictive maintenance strategies that covered the entirety of their manufacturing processes. Rather, when predictive maintenance was found, it was in isolated instances at the machine or component level.

Technology providers illuminated the fact that their only motivation to incorporating PHM technologies into their manufacturing solutions was if it was required to satisfy specific customer requirements. Granted, added levels of PHM increase the cost of the overall solution where some manufacturers pushed back against higher costs. In turn, the technology providers noted that achieving a lower cost called for stepping down the capabilities of the system. The manufacturers had to weigh whether or not the added the cost of the PHM solution was worth the investment.

### 4.2. Maintenance Factors: Comparison between SMEs and Large-Sized Manufacturers

This section focuses on comparing the level of development of intelligent maintenance technologies and the strategies between SME manufacturers and large-sized manufacturers from various aspects. This study has identified eight key factors related to maintenance based on the questionnaire results. Each factor is scored on a 0 % – 100 % scale, where 66.7 % – 100 % represents the most advanced level in terms of performance and effectiveness (level 3), 33.3 % – 66.7 % represents the intermediate level (level 2), and 0 % – 33.3 % corresponds to the beginning level which is least intelligent in maintenance technology and strategy as well as their effectiveness (level 1). Table 5 in the Appendix of this paper defines the levels for each of the eight factors.

**Figure 5:**
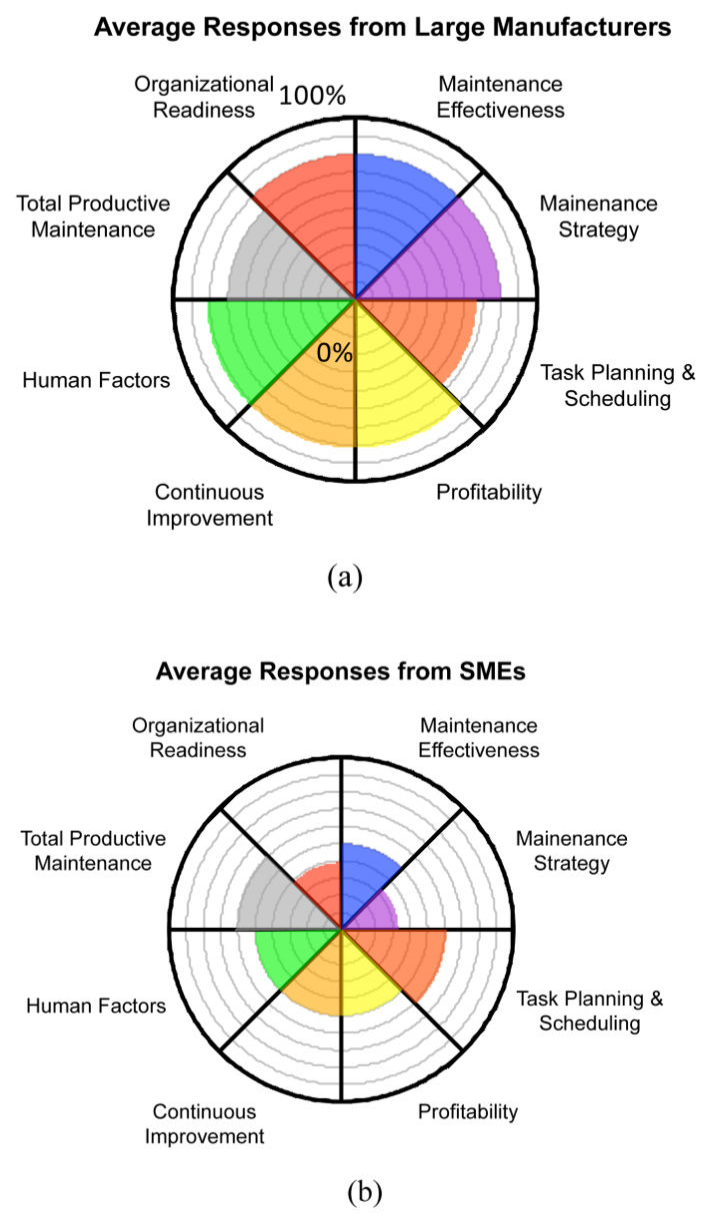
Radar charts for manufacturing enterprises with different sizes: (a) large-sized manufacturing enterprises, (b) SMEs

The responses to the interval questions are averaged and plotted in radar charts for large-sized enterprises and SMEs, respectively, to study how enterprise size may influence these key factors of maintenance. According to the responses to the interval questions based on Table 5, the average levels of eight key factors are presented for large firms and SMEs in Figure 5.

### 4.3. Correlation Analysis for Maintenance Factors

The survey responses, as obtained by UC/UM, can be seen as ordinal data; thus, correlation analysis is adopted. The Spearman’s rank correlation and Kendall’s tau correlation are both recommended for the analysis of ordinal data (Muchiri et al., 2010). Therefore, both the Spearman’s rank correlation and Kendall’s tau correlation are adopted.

The Spearman’s rank correlation coefficient is defined as the Pearson correlation coefficient [Edwards, 1976] between the ranked variables. For a sample of size *n*, then raw scores *X_i_*, *Y_i_* are converted to ranks *x_i_*, *y_i_*, and the Spearman correlation coefficient *ρ* is computed as:

(3) $$\rho =1-\frac{6\sum_{i=1}^{n}d_{i}^{2}}{n(n^{2}-1)}$$

where *d_i_* is the difference between the two ranks of each observation and *n* is the number of observation [Daniel, 1990].

The results of Spearman correlation analysis for eight maintenance-related factors are presented in Table 6. The Kendall’s tau correlation is a measure of rank correlation: the similarity of the orderings of the data when ranked by each of the quantities. The results of Kendall’s tau correlation analysis are presented in Table 7.

It was found that maintenance effectiveness, maintenance strategy, profitability, continuous improvement, human resources for maintenance, and organizational readiness are significantly correlated with the size of the manufacturing enterprise. In particular, the organizational readiness is highly correlated with maintenance strategy and company size (*p*<=0.01 in both Spearman’s correlation and Kendall’s tau correlation). It is also significantly correlated with human resources for maintenance and continuous improvement (with *p*<=0.05 in Spearman’s correlation). However, the correlations between Scheduling, Total Productive Maintenance (TPM) and Size are not significant.

### 4.4. Correlation Analysis: PHM and Size of Manufacturers

The correlation analysis indicates that a relationship exists between the size of the manufacturing enterprise and the eight key factors. A statistic test is adopted to do the hypothesis testing to see whether the differences between SMEs and large-sized manufacturers are statistically significant.

Due to the small sample number, the Student’s *t* test is used to check whether there are significant differences in each factor between large manufacturers and SMEs. All eight factors in Table 5 are tested between SMEs and large-sized manufacturers. Two examples of maintenance strategy level and scheduling level for SMEs versus large firms is presented below to explain how the hypothesis test works.

#### Example 1: Maintenance Strategy Level

The null hypothesis on maintenance strategy level H_0_ is that the mean maintenance strategy level of SMEs equals the mean maintenance strategy level of large-sized manufacturers. The results of a Student’s t-test are shown in Table 8 in Appendix.

Levene’s test is used to check whether the variances of two groups are equal because Levene’s test is an inferential statistic used to assess the equality of variances for a variable calculated for two or more groups. The significance of F-value is 0.023, which is less than 0.05, meaning that the variances in the two groups are not equal, i.e., equal variance is not assumed. According to the T table, two-tailed *t*(0;05,9) is less than the absolute t-value, i.e., |*t*| > *t*(0.05,9). Therefore, H_0_ is rejected, indicating that the mean maintenance strategy level of large-sized manufacturers is significantly larger than the mean maintenance strategy level of SMEs.

#### Example 2: Scheduling level

H_0_: The mean scheduling level of SMEs equals the mean scheduling level of large-sized manufacturers.
H_a_: The mean scheduling levels of SMEs and large-sized manufacturers are different.

The Levene’s test result, the significance of F-value, 0.362, which is greater than 0.05, so the variances of two groups are assumed to be equal; thus, |*t*|=−1.028 < *t*(0.05,11), so accept H_0_. It can be concluded that the mean scheduling levels of large-sized manufacturers and SMEs are equal. The results of Student’s t test are shown as in Table 9 in Appendix.

From the hypothesis testing results for all eight factors, the key findings can be summarized as follows: large manufacturers, in contrast to SMEs, have the ability to focus on two distinct strategies: 1) continuous improvement on condition-based maintenance and/or predictive maintenance technology and level of sophistication, and 2) a combination of low-cost maintenance technology and strategy innovation.

## 5. Gaps, Future Trends, and Research Directions

We identify some gaps, challenges and future trends for manufacturing PHM and maintenance strategy based on the observations from the case studies and statistical analysis of the data collected from survey respondents. The overall state of the art for manufacturing PHM has many current gaps, which can be divided into two categories: (1) maintenance strategy levels, and (2) diagnostics/prognostics technologies.

Maintenance strategy levels are relatively low in most manufacturing enterprises. These levels range from reactive maintenance to preventive maintenance (time-based or cycle-based maintenance). Very few predictive or proactive maintenance practices were adopted by the surveyed manufacturing enterprises. The common barriers that inhibit these manufacturers to improve their maintenance strategies are mainly costs, workforce and level of skills, organizational and technology readiness, and complexity of system design changes. In addition, compared with SMEs, large manufacturing enterprises are making more efforts to improve their maintenance strategy because of their size-related advantages such as R&D support, leadership involvement, skilled workforce and other resources. In addition, having a clear strategy on how to motivate and train plant personnel on this technology and take appropriate action from these diagnostic and prognostic alerts should not be overlooked.

Diagnostic and prognostic technologies implemented in most of the manufacturing enterprises have been limited to component and machine level fault detection diagnosis. There are very few system-level diagnostics and prognostics implementations that support multiple components interacting within a production system. Although some research has been looking at system-level health monitoring and assessment, very few successful implementations have been found in real applications due the complex interdependencies among components and subsystems within a manufacturing system. Some technology providers are making more efforts to develop system level health monitoring system and PHM by using large amount of data collected from sensors, controllers and automation systems in the plant floor to monitor or predict system health. In addition, even current component-level and machine-level prognostics and diagnostics techniques lack robustness and adaptiveness, thus limiting their successful implementation by manufacturers. Common issues noted by the manufacturers include unsatisfactory number of false alarms, and difficulty in setting up baseline conditions for fault detection and diagnosis that consider various operating conditions. It was also noted by the technology providers that the lack of failure data makes it more challenging to develop robust prognostic and diagnostic methods. Furthermore, without reference data sets that include failure data, validation of the technology becomes very difficult. Gaps and barriers for implementing advanced PHM technologies identified in the study are also well aligned with the findings of the 2015 NIST PHM workshop report (NIST, 2015).

Based on these findings and current gaps, future research needs and directions should focus on the development of new technologies and infrastructure to support PHM system implementation for smart manufacturing. One important step for industry, to move from a fail-and-fix paradigm to a predictive-and-proactive paradigm, is to create incentives and evolve the culture, so they can change from passive to proactive maintenance and operations. Interdepartmental collaboration, R&D support and leadership will also be primary to the success of the paradigm shift. Other future PHM research and technology development would include more system-level diagnostics and predictive analytics by fully utilizing both engineering knowledge and industrial big data, as well as automated decision-making for maintenance scheduling and operations planning.

## 6. Conclusions and Future Work

This paper conducted a comprehensive study to investigate the best practices that the United States manufacturing enterprises are currently using to achieve their performance goals by incorporating both diagnostic and prognostic technologies and maintenance strategies. With that notion in mind, data was collected by phone interviews and on-site facility visits from various manufacturing enterprises, including a total of fifteen manufacturing enterprises and eight technology/consulting companies. NIST also provided additional case studies of SMEs to complement the survey-based study during site visits and factory tours of several manufacturing and technology integration facilities. While the UC/UM team analyzed the detailed survey data, both teams (UC/UM and NIST) assessed the information from the conversational case studies. This team-based approach allowed UC/UM and NIST to jointly formulate what they see as the future directions in PHM given the identified gaps and issues.

One of the interesting findings during this study was that the maintenance effectiveness, maintenance strategy, and human resources for maintenance were significantly correlated with the size of the manufacturing enterprise. There was an obvious difference in maintenance technology and strategy when comparing large and small/medium manufacturing enterprises. Even for the larger manufacturing enterprises, it was noted that the effectiveness of their intelligent maintenance programs varied between the different organizations. Many organizations had mixed success with respect to their past diagnostic and prognostic projects. Despite this mixed level of success, many of the manufacturing organizations surveyed had active diagnostic and prognostic projects and had an overwhelming positive and optimistic viewpoint when considering the future outlook for manufacturing PHM.

The results from this study illustrate many future research directions to address the gaps identified in this study. The literature review highlighted a sparse set of technical work on system-level PHM for factory applications, in comparison to the machine-level and component-level PHM work for robotics, machine tools, and other manufacturing equipment; thus the need to develop technical approaches for system-level PHM for factory applications is one potential future research direction. In addition, some manufacturers were unsatisfactory in the threshold setting and overall robustness in the PHM machine-level models; this reiterates that there is a still a need to improve the current state of the art with respect to PHM for manufacturing components and machines. Lastly, there is a significant gap between SME and large manufacturing organizations, in which the SME would benefit from at least learning from the large manufacturers and their early trials and success with PHM and maintenance technology. With this notion, it would be beneficial to make a concerted effort to disseminate the PHM manufacturing case studies, with the aim that SMEs would eventually consider adopting these maintenance technologies with a good fit.

Besides the gaps and issues being identified in this study, many other challenges and barriers that prevent manufacturers from adopting advanced PHM technologies will be further explored and discussed in the future work, such as the need for using digital technologies for data collection and handling and interpreting “industrial big data,” the need to develop protocols and tools to communicate data, information and metrics across the component, machine and system levels for diagnostics and prognostics in manufacturing, and the need to enhance operations and maintenance intelligent by predictive and preventive control and management.

## Figures and Tables

**Table 1:** Maintenance Strategy Characteristics

Maintenance Strategy	Reactive Maintenance (RM)	Preventive Maintenance (PM)	Predictive Maintenance (PdM)	Proactive Maintenance (PaM)
**Maintenance Interval**	Fail-and-fix	Time based; Usage based	Reliability based; Condition based	Improve & sustain
	
**Object**	Component; Sub-system; System	Component; Sub-system; System	Component; Function; System	Component; Function; System
	
**Planning & Scheduling**	Planning on the fly	Planning & scheduling based on ideal PM interval	Predictive planning & scheduling	Proactive planning & scheduling
	
**Human Factors (inspection & decision-making)**	Medium to High	Intermediate	Low	Low (false alarm)
	
**Cost Effectiveness**	Labor intensive; Labor and material	Costly due to over maintenance or ineffective & inefficient PM	Cost-effective; extended life & less failure- induced costs	Cost-effective: Substantially save failures & extend the life of equipment
	
**Requirement for Technology Readiness**	Low	Low to Medium	High	High

**Table 2:** Participating enterprises segmented by size & type

	SME	Large	Total	Percent
**Manufacturing Enterprise**	3	12	15	65.2%
**Technology/Consulting Enterprise**	5	3	8	34.8%
**Total**	8	15	23	100%

**Table 3:** Chi-Square Test Results – Failure Mode/Criticality Analysis Response

cχ^2^	5.5
α	0.05
df	3
p-value	0.1386
Hypothesis	H_0_

**Table 4:** Chi-Square Test Results – Manufacturing PHM Trend Optimism

cχ^22^	11.6
α	0.05
df	3
p-value	0.0089
Hypothesis	H_a_

## Footnotes

1 Burn Rate is the rate at which an enterprise spends money, especially venture capital, in excess income.

## Appendix

**Table 5:** Key factors versus maintenance performance at various levels

Factors	Level 3 (100%) Advanced (predictive & proactive)	Level 2 (66.7%) Intermediate (preventive)	Level 1 (33.3%) Beginning (reactive)
*Maintenance Effectiveness*	Maintenance performance is very satisfactory where no improvement is warranted.	Maintenance program is effective but could still be improved.	Maintenance has significant room for improvement, or Preventive maintenance program is lacking/reactive maintenance
	
*Maintenance Strategy*	Employ predictive maintenance (PdM) strategy for sustainable improvement. All problems are analyzed and permanently solved. Reactive maintenance is minimized.	Use preventive maintenance (PM) as a main approach, usually age-based or cycle-based. Some reactive maintenance is required.	Rely heavily on reactive maintenance (RM), no equipment health information involved
	
*Task Planning and Scheduling*	More than 90 % of work that is planned is accomplished. Low overtime for maintenance activities (<15 %)	More than 50 % work planned accomplished. Relatively high overtime ( >15 %)	Less than 50 % work planned accomplished. High overtime ( >30 %)
	
*Profitability*	Significant cost savings due to failure reduction and life extension	Cost-effectiveness is satisfactory	Not cost-effective
	
*Continuous improvement*	Proactive maintenance. CBM or PHM applied, performance measurements are in place and effectively used	Have preventive maintenance in place with management involved in policy settings and reviews	Have no CBM or PHM. Low involvement of management. Reactive maintenance is very common
	
*Maintenance Training (Human factors)*	Educational plans are designed for each maintenance worker. A global R&D Team is in place that is responsible for developing and implementing prognostic and diagnostic techniques	Skilled staff normally qualified on a few machines. A small team is in place that is responsible for developing and implementing prognostic and diagnostic techniques	No training on how to use maintenance strategies. Lack of system to collect maintenance knowledge. No team that is responsible for developing and implementing prognostic and diagnostic techniques
	
*Total productive performance (TPM)*	Overall Equipment Effectiveness (OEE) is greater than 80 %	Overall Equipment Effectiveness (OEE) is between 50% and 80%	Overall Equipment Effectiveness (OEE) is less than 50%
	
*Organizational Readiness*	Leadership Involvement and strong R&D support	Lack of sufficient R&D support & leadership involvement	“Fire Fighting” approach

**Table 6:** The Spearman correlation of different maintenance factors

		F1	F2	F3	F4	F5	F6	F7	F8	F9
		Size	Effect.	Strategy	Sched.	Profitability	Impr.	HR	TPM	Readiness
F1	Size	1.000	.663*	.758**	.298	.660*	.670*	.660*	.463	.755**
F2	Effectiveness	.663*	1.000	.199	.224	.870**	.728**	.762**	.491	.370
F3	Strategy	.758**	.199	1.000	.237	.341	.544	.436	.259	.902**
F4	Scheduling	.298	.224	.237	1.000	.085	-.053	-.085	.033	.190
F5	Profitability	.660*	.870**	.341	.085	1.000	.719**	.905**	.256	.499
F6	Improvement	.670*	.728**	.544	-.053	.719**	1.000	.864**	.401	.732**
F7	HR	.660*	.762**	.436	-.085	.905**	.864**	1.000	.256	.593*
F8	TPM	.463	.491	.259	.033	.256	.401	.256	1.000	.279
F9	Readiness	.755**	.370	.902**	.190	.499	.732**	.593*	.279	1.000

* The correlation is significant at the level of 0.05 (two-sided)

** The correlation is significant at the level of 0.01 (two-sided)

**Table 7:** The Kendall’s tau correlation of different maintenance factors

		F1	F2	F3	F4	F5	F6	F7	F8	F9
		Size	Effect.	Strategy	Sched.	Profitability	Impr.	HR	TPM	Readiness
F1	Size	1.000	.608*	.696*	.290	.603*	.613*	.603*	.452	.685**
F2	Effectiveness	.608*	1.000	.130	.204	.829**	.678**	.699**	.453	.320
F3	Strategy	.696**	.130	1.000	.202	.258	.443	.339	.225	.857**
F4	Scheduling	.290	.204	.202	1.000	.090	-.046	-.090	.031	.155
F5	Profitability	.603*	.829**	.258	.090	1.000	.656**	.871**	.225	.413
F6	Improvement	.613*	.678**	.443	-.046	.656**	1.000	.820**	.365	.613*
F7	HR	.603*	.699**	.339	-.090	.871**	.820**	1.000	.225	.492*
F8	TPM	.452	.453	.225	.031	.225	.365	.225	1.000	.243
F9	Readiness	.685**	.320	.857**	.155	.413	.613*	.492*	.243	1.000

* The correlation is significant at the level of 0.05 (two-sided)

** The correlation is significant at the level of 0.01 (two-sided)

**Table 8:** Student’s t-test for maintenance strategy comparison between large enterprises and SMEs

	Levene’s test for equality of variances		t-test for equality of means		

	*F*	Significance	*t*	*df*	Significance (2-tailed)
Equal variance assumed	6.913	0.023	−5.961	11	0.000094
Equal variance not assumed			−11.225	9	0.000001

**Table 9:** Student’s t-test for scheduling level comparison between large enterprises and SMEs

	Levene’s test for equality of variances		t-test for equality of means		

	*F*	Significance	*t*	*df*	Significance (2-tailed)
Equal variance assumed	0.906	0.362	−1.028	11	0.326
Equal variance not assumed			−0.913	2.855	0.432
